# Modulation of Autophagy by Oncosuppressor FAM46C and Its Implications for Cancer Therapy: An Intriguing Perspective

**DOI:** 10.3390/biom15020196

**Published:** 2025-01-30

**Authors:** Nicola Manfrini

**Affiliations:** 1 INGM, Istituto Nazionale Genetica Molecolare Romeo ed Enrica Invernizzi, 20122 Milan, Italy; nicola.manfrini@unimi.it; 2 Department of Biosciences, University of Milan, 20133 Milan, Italy

**Keywords:** TENT5C, tumour suppressor, multiple myeloma, autophagy

## Abstract

Cancer is one of the major challenges in medicine, necessitating continuous advancements in therapeutic approaches. Autophagy, an intracellular pathway essential for cellular homeostasis and stress response, has emerged as a promising target for cancer treatment. In this context, FAM46C, a novel pan-cancer tumour suppressor, has been shown to induce apoptosis in multiple myeloma cells through indirect inhibition of autophagy. Here, we discuss how FAM46C-induced autophagic dampening could offer new opportunities for global cancer therapy. Specifically, we explore two scenarios in which the expression of a functional FAM46C may either sensitize cancer cells to autophagic inhibition or antagonize their sensitivity. We further comment on how this synergism/antagonism could be used to refine strategies for cancer treatment, positioning FAM46C as a pivotal factor in future cancer therapy development.

## 1. Introduction

To date, cancer remains one of the biggest challenges in medicine and, thus, necessitates the continuous development of therapeutic strategies.

Given that cancer cells demand an abnormal quantity of nutrients to foster their uncontrolled proliferation, targeting either growth/metabolic pathways or mechanisms involved in preserving cell function/homeostasis has been, for decades, the most efficient way to cope with cancer disease.

Autophagy is a key intracellular process responsible for (1) proper degradation and recycling of cellular components, (2) maintenance of cellular homeostasis, and (3) insurance of proper cellular function during periods of stress.

At the molecular level, autophagy relies on the formation of autophagosomes, double-membrane vesicles that engulf cellular components and fuse with lysosomes in order to elicit their degradation.

Physiologically, autophagy is required for cell survival. However, its activity has to be fine-tuned, as uncontrolled hyper-activation of autophagy can be highly deleterious and trigger cell demise [1].

In cancer, the role of autophagy is bi-directional. On one hand, autophagy prevents tumour initiation and malignant transformation by clearing damaged mitochondria, in turn reducing oxidative stress and consequent genomic instability, critical events for tumorigenesis. On the other hand, as the tumour develops and progresses, usually in hypoxic and nutrient-deprived environments, autophagy is triggered to sustain the increased metabolic demand of highly proliferative cancer cells [1]. Hence, despite autophagy’s important role in preventing tumour onset, its pro-survival role in established cancer cells makes autophagic inhibition a clinical approach that is gaining increasing attention from the scientific community [2].

Currently, two autophagic inhibitors, chloroquine (CQ) and hydroxychloroquine (HCQ), which block lysosomal acidification and consequently its degradatory potential, have been approved by the FDA, and autophagic targeting has been shown to efficiently augment standard chemotherapy and target therapy [3]. In line with this evidence, the identification of novel inhibitors of the autophagic pathway is well underway, with plans to provide novel putative autophagy-targeting drugs in the near future [3].

Generally speaking, however, not all cancer cells are equally dependent on autophagy [4] and, therefore, should not be equally sensitive to autophagy inhibitors. Determining in advance the sensitivity of a cell to autophagic inhibition and identifying the specific features that might render it more or less sensitive could lead to important advancements in cancer treatment.

FAM46C is a tumour suppressor gene originally characterized in multiple myeloma (MM) [5], but its antitumoural function is now being established for a broad range of cancer types, attracting significant interest from the scientific community [6,7].

Despite its mode of functioning still being debated and different models being proposed to try to explain it [7], clear evidence indicates that FAM46C modulates autophagic functionality. Specifically, our group has shown that in MM cells, which are highly-secreting malignant plasma cells, FAM46C can inhibit autophagy through alteration of intracellular trafficking dynamics [5]. In such a scenario, where cells are already highly stressed by endoplasmic reticulum overload, autophagic inhibition triggers intracellular protein aggregate accumulation and consequent apoptosis induction, providing an explanation for FAM46C tumour suppressor activity.

Interestingly, the autophagic inhibition triggered by FAM46C is not restricted to the specific MM environment. In fact, in the lab, we have recently shown that by inhibiting autophagy in HEK-293T cells, FAM46C is capable of restraining lentiviral particle production [8].

Such a result indicates that FAM46C-induced inhibition of autophagy is a more general and non-cell-specific occurrence.

Given the emerging involvement of FAM46C as a tumour suppressor in multiple cancer types [7], the autophagic inhibition induced by FAM46C might offer new opportunities for global cancer therapy implementation.

## 2. Possible Effects of FAM46C Expression on the Efficacy of Cancer Treatment with Autophagy Inhibitors

**Figure 1:**
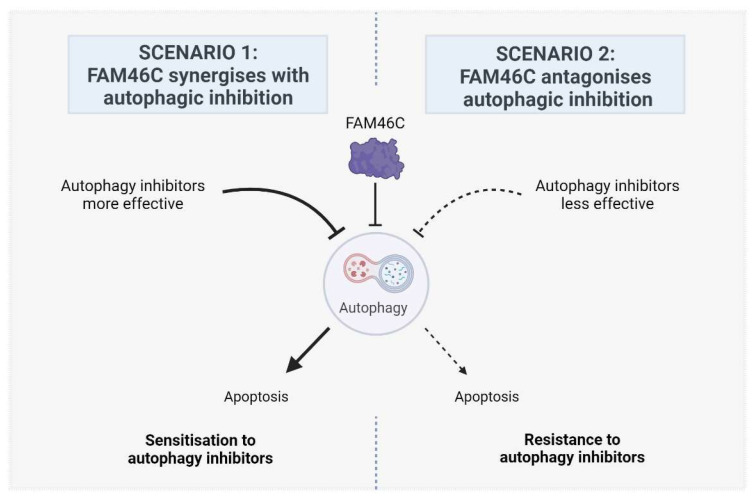
Proposed models describing the possible effects of FAM46C expression on the efficacy of cancer treatment with autophagy inhibitors. In scenario 1, FAM46C is expected to synergise with autophagic inhibition, rendering cells more sensitive to the administration of autophagy inhibitors. In scenario 2, FAM46C is expected to antagonise autophagic inhibition, rendering cells less sensitive to the administration of autophagy inhibitors. Image created with BioRender.com.

Despite several studies showing that the presence or absence of FAM46C expression can affect the efficacy of numerous anticancer drugs in different tumour environments [7], inhibitors of autophagy have not yet been tested. However, we can predict with awareness that expression of a functional FAM46C may either (1) augment or (2) antagonize autophagic inhibition (Figure 1).

### 2.1. Scenario 1: FAM46C Synergizes with Autophagic Inhibition

The first scenario (Figure 1, Left) relies on the fact that concurrent inhibition of the same pathway by both FAM46C and autophagy inhibitors may lead to synergistic outcomes and is supported by numerous indirect pieces of evidence:

- The combined action of dexamethasone, a corticosteroid with anti-inflammatory and anti-angiogenesis effects, with andrographolide, a botanical with antitumourigenic properties, was shown to inhibit autophagy [9] and several studies found that FAM46C expression synergizes with dexamethasone administration [7].
- Recently, the administration of thalidomide, an immunomodulatory drug used for MM treatment, was shown to correlate with decreased accumulation of intracellular LC3B [10], an autophagic marker, and FAM46C expression was shown to synergize with the administration of lenalidomide, a thalidomide analogue [11].
- SK1-I, a sphingosine kinase inhibitor with a strong antiproliferative and pro-apoptotic activity, was shown to trigger autophagy, but impair autophagic flux [12], and FAM46C expression was shown to be synergistic with SK1-I administration [5].
- Docetaxel, a chemotherapeutic that was shown to synergize with FAM46C expression [13], was recently shown to be capable of inhibiting autophagy through a mechanism which requires the presence of MYH9 [14], a known protein interactor of FAM46C [8].
- Bortezomib, a common proteasome inhibitor used for MM therapy, was shown to inhibit autophagy in cancer cells [15] and, recently, MM cells with low FAM46C levels were shown to be less sensitive to bortezomib administration compared to those with higher FAM46C levels [16].
- Lastly, doxorubicin, an anthraquinone chemotherapeutic agent, was shown to negatively regulate autophagy [17], and FAM46C knockout was shown to render cells insensitive to doxorubicin administration [18].

By exploring the drug sensitivity of MM cell lines, either lacking (LP-1, OPM-2) or expressing high levels of a functional FAM46C protein (MM1.S, RPMI-8226) [5], using the Genomics of Drug Sensitivity in Cancer (GDSC) repository (https://www.cancerrxgene.org/), we confirmed, at least in part, this scenario. Focusing only on drugs with an inhibitory effect on autophagy, we found that high levels of FAM46C correlate with significant sensitisation to dihydrorotenone, AZD5991, or LMB_AB2, drugs that target mitochondrial complex I, MCL1 and GADD34, respectively, but that were also predicted to associate with autophagic inhibition [19,20,21] and confirmed that the absence of FAM46C is associated with resistance to doxorubicin administration (Table 1).

### 2.2. Scenario 2: FAM46C Antagonises Autophagic Inhibition

The second scenario (Figure 1, Right), is based on the idea that if autophagy is less active in cells expressing a functional FAM46C, it might also be less efficiently targeted/targetable. In line with this vision, autophagy might be conversely more targetable in cells lacking a functional FAM46C.

Although no published data currently directly support this model, exploration of the GDSC repository revealed that lack of FAM46C in MM cells is indeed associated with sensitivity to drugs shown to indirectly inhibit autophagy, namely avagacestat [22] and, again, dihydrorotenone [19] and AZD5991 [20] (Table 1).

Overall, the general indication is that either lack or high levels of FAM46C may affect cell sensitivity to autophagic inhibition.

However, all available evidence is based solely on sensitivity to drugs indirectly inhibiting autophagy, making it difficult to draw any final conclusion on FAM46C’s impact on direct autophagic targeting.

Given that the oncosuppressive roles of FAM46C, including inhibition of autophagy, rely on its interaction with the FNDC3 proteins, namely FNDC3A [5] and FNDC3B [23], we explored whether there was any association between FNDC3A and/or FNDC3B levels and the sensitivity of MM cell lines to direct autophagic inhibitors. Again, we took advantage of the GDSC repository and focused only on those MM cell lines having either high (MOLP-8) or low (KMS-12-BM) levels of both FNDC3A and FNDC3B (as annotated by the human protein atlas; https://www.proteinatlas.org/).

Notably, MOLP-8 cells were sensitive to direct autophagy inhibitors, namely VSP34_8731, a VSP34 inhibitor, and ULK1_4989, a ULK1 inhibitor, whereas KMS-12-BM cells were not (Table 2). Furthermore, MOLP-8 cells exhibited the highest overall sensitivity to these drugs among all cell lines in the GDSC repository (https://www.cancerrxgene.org/).

Altogether, these results suggest a correlation between high FNDC3 levels and sensitivity to direct autophagic inhibitors and would pinpoint the first scenario (Figure 1, Left) as the most likely to occur.

## 3. Future Directions

In general, despite these preliminary correlations, more focused studies using other canonical, direct autophagy inhibitors are necessary to determine which scenario is most probable and to define the extent of the synergism/antagonism between FAM46C expression and the efficacy of autophagy inhibitors. Once this is determined, it would be tempting to be able to stratify patients based on FAM46C/FNDC3A expression levels or mutational status, as this could enable us to predict cancer responsiveness to autophagy inhibitors and enable tailored and personalised therapies.

Here, we focused only on drugs known to inhibit autophagy, not on those that induce it. However, future analyses should also consider autophagic inducers, as synergism/antagonism might occur with these drugs as well. Again, we would expect two possible scenarios: one, in which high FAM46C levels might dampen the effects of autophagic induction, and the other, where high FAM46C levels could enhance the efficacy of autophagic inducers. In either case, the results will be of fundamental importance for therapy implementation.

Last, but not least, defining the exact molecular mechanisms through which FAM46C is inhibiting autophagy and triggering apoptosis becomes of fundamental importance, as it would facilitate the selection of drugs to be screened for synergistic or antagonistic interactions with FAM46C expression.

## 4. Conclusions

In conclusion, targeting autophagy in cancer therapy represents a promising strategy to improve the clinical outcome of patients with various types of cancer.

The emerging role of pan-cancer tumour suppressor FAM46C as a regulator of autophagy adds a new dimension to this therapeutic approach and requires consideration in future research and clinical applications.

## Figures and Tables

**Table 1:** Drugs associated with autophagic inhibition for which the listed cell lines are either significantly sensitive or significantly resistant. A total of four MM cell lines with either lack (LP-1 and OPM-2) or high levels (MM1-S and RPMI-8226) of a functional FAM46C were analysed. The outcome induced by drug administration in the specific cell type was associated with a specific scenario: 1 or 2 (please refer to Figure 1). Data were retrieved from the GDSC database (https://www.cancerrxgene.org/) using both the GDSC1 and GDSC2 datasets. Z-scores ≥2 or ≤−2 were considered significant. References related to the autophagic inhibition induced by each drug are listed.

MM Cell Line	Wt FAM46C Status	Drug	Outcome	Effect on Autophagy	Scenario	Reference
MM1.S	High levels	Dihydrorotenone	SENSITIVITY	Inhibition	1	[19]
RPMI-8226	High levels	AZD5991	SENSITIVITY	Inhibition	1	[20]
	High levels	LMB_AB2	SENSITIVITY	Inhibition (predicted)	1	[21]
LP-1	Absence	Avagacestat	SENSITIVITY	Inhibition	2	[22]
	Absence	Dihydrorotenone	SENSITIVITY	Inhibition	2	[19]
	Absence	AZD5991	SENSITIVITY	Inhibition	2	[20]
	Absence	Doxorubicin	RESISTANCE	Inhibition	1	[17]
OPM-2	Absence	AZD5991	SENSITIVITY	Inhibition	2	[20]
	Absence	Doxorubicin	RESISTANCE	Inhibition	1	[17]

**Table 2:** Drugs associated with direct autophagic inhibition for which the listed cell lines are either significantly sensitive or non-sensitive. Two MM cell lines with either high (MOLP-8) or low (MKS-12-BM) levels of FNDC3A and FNDC3B were analysed. The outcome induced by drug administration in the specific cell type was associated with a specific scenario: 1 or 2 (please refer to Figure 1). Data were retrieved from the Genomics of Drug Sensitivity in Cancer database (https://www.cancerrxgene.org/) using both the GDSC1 and GDSC2 datasets. Z-scores ≥2 or ≤−2 were considered significant.

MM Cell Line	FNDC3A/ FNDC3B Status	Drug	Target	Outcome	Scenario
MOLP-8	High levels	VSP34_8731	VSP34	SENSITIVITY	1
	High levels	ULK1_4989	ULK1	SENSITIVITY	1
MKS-12-BM	Low levels	VSP34_8731	VSP34	NON-SENSITIVITY	1
	Low levels	ULK1_4989	ULK1	NON-SENSITIVITY	1
